# Marine-Fungi-Derived Gliotoxin Promotes Autophagy to Suppress *Mycobacteria tuberculosis* Infection in Macrophage

**DOI:** 10.3390/md21120616

**Published:** 2023-11-28

**Authors:** Jun Fu, Xiaowei Luo, Miaoping Lin, Zimin Xiao, Lishan Huang, Jiaxi Wang, Yongyan Zhu, Yonghong Liu, Huaming Tao

**Affiliations:** 1 School of Traditional Chinese Medicine, Southern Medical University, Guangzhou 510515, China; fjun99alyssaaa@163.com (J.F.);; 2 Guangxi Key Laboratory of Marine Drugs, Institute of Marine Drugs, Guangxi University of Chinese Medicine, Nanning 530200, China

**Keywords:** marine natural product, gliotoxin, *Mycobacterium tuberculosis* (MTB), macrophages, autophagy

## Abstract

The *Mycobacterium tuberculosis* (MTB) infection causes tuberculosis (TB) and has been a long-standing public-health threat. It is urgent that we discover novel antitubercular agents to manage the increased incidence of multidrug-resistant (MDR) or extensively drug-resistant (XDR) strains of MTB and tackle the adverse effects of the first- and second-line antitubercular drugs. We previously found that gliotoxin (**1**), 12, 13-dihydroxy-fumitremorgin C (**2**), and helvolic acid (**3**) from the cultures of a deep-sea-derived fungus, *Aspergillus* sp. SCSIO Ind09F01, showed direct anti-TB effects. As macrophages represent the first line of the host defense system against a mycobacteria infection, here we showed that the gliotoxin exerted potent anti-tuberculosis effects in human THP-1-derived macrophages and mouse-macrophage-leukemia cell line RAW 264.7, using CFU assay and laser confocal scanning microscope analysis. Mechanistically, gliotoxin apparently increased the ratio of LC3-II/LC3-I and Atg5 expression, but did not influence macrophage polarization, IL-1β, TNF-a, IL-10 production upon MTB infection, or ROS generation. Further study revealed that 3-MA could suppress gliotoxin-promoted autophagy and restore gliotoxin-inhibited MTB infection, indicating that gliotoxin-inhibited MTB infection can be treated through autophagy in macrophages. Therefore, we propose that marine fungi-derived gliotoxin holds the promise for the development of novel drugs for TB therapy.

## 1. Introduction

Tuberculosis (TB) caused by the *Mycobacterium tuberculosis* (MTB) infection remains a devastating global health problem. Although, MTB adopts a successful strategy for persisting in the host and circumventing host defense; therefore, burgeoning drug resistance of TB remains threateningly high [1]. In addition, since traditional anti-TB treatment regimens function takes an extremely long time together with side effects [2], it is urgent that we discover and develop effective host-directed therapy with minimal side effects to cure TB. At present, various approved drugs, especially antibacterial agents, have been developed from microbial natural products [3]. The deep sea, with its unique aquatic environment and plentiful biodiversity, is a rich source of natural products with antitubercular properties [4]. Marine-derived fungi, especially, *Aspergillus* and *Penicillium*, have been continuously evidenced as producers of diverse chemical structures with anti-inflammatory and antitubercular properties [5,6]. However, further investigation is needed to clarify whether these putative new compounds are essential for anti-TB effects under in vitro conditions.

Macrophages elicit an early innate immunity and represent the first line of host defense to anti-mycobacterial responses [7,8]. During MTB infection, the functional and morphologic heterogeneity of macrophages play a pivotal role in host–pathogen interactions [9]. M1-like macrophages produce pro-inflammatory cytokines, oxygen free radicals, including tumor necrosis factor alpha (TNF-α), interleukin 1 beta (IL-1β), interleukin 6 (IL-6), and reactive oxygen species (ROS), as well as autophagy; therefore, they possess many microbicidal properties and play a vital role in Mtb clearance in macrophages. M2-like macrophages are associated with anti-inflammatory cytokines secretions, such as interleukin-10 (IL-10) [10], and reduction of autophagy, leading to Mtb proliferation [11,12]. Importantly, autophagy acts as a host defensive strategy, resulting in phagosomal maturation that mediates mycobacteria clearance [13]. In fact, there is a study that has developed numerous synthetic compounds targeting autophagy to enhance antimicrobial defense against MTB infection [14]. Thus, an understanding of the mechanism of action of putative new agents for TB treatment in host macrophages will facilitate the development of new drugs for TB therapy.

In our previous study, 12 bioactive compounds were obtained from the cultures of the deep-sea-derived fungus *Aspergillus* sp. SCSIO Ind09F01. Among them, gliotoxin (**1**), 12, 13-dihydroxy-fumitremorgin C (**2**), and helvolic acid (**3**) exhibited direct anti-tuberculosis effects [5]. However, how these compounds counteract with MTB infection in the host’s immune defense system remains unknown. Here, we further demonstrate that gliotoxin exerts a potent anti-tuberculosis effect in human and mice macrophages. Moreover, we reveal that gliotoxin inhibits MTB infection through autophagy in macrophage. Therefore, we propose that a marine-fungi-derived gliotoxin could be a potential lead compound for the development of anti-TB drugs.

## 2. Results

### 2.1. Gliotoxin Inhibits MTB Infection in THP-1-Derived Macrophages

**Figure 1:**
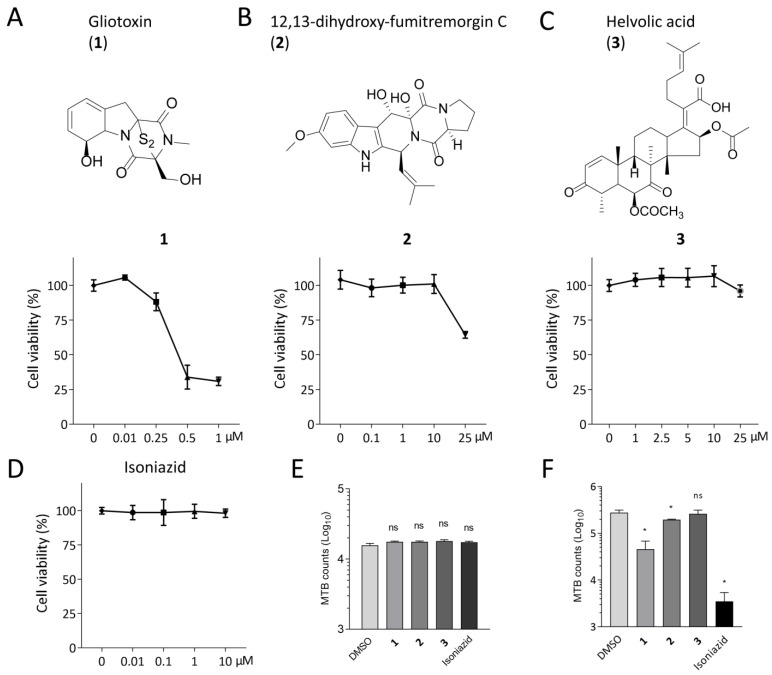
Gliotoxin inhibited MTB infection in THP-1-derived macrophages. (**A**–**C**) Chemical structures of marine-fungi-derived gliotoxin (**1**), 12, 13-dihydroxy-fumitremorgin (**2**), and helvolic acid (**3**) were shown and CCK8 kit was used to detect cytotoxicity of THP-1-derived macrophages with treatment of different concentrations of these compounds for 48 h. (**D**) CCK8 assay was applied to detect cell proliferation of THP-1-derived macrophages with treatment of different concentrations of isoniazid for 48 h. (**E**,**F**) CFU assay has been determined for MTB survival with treatments of 0.25 μM gliotoxin, 10 μM 12, 13-dihydroxy-fumitremorgin C, 25 μM helvolic acid, and 10 μM isoniazid in THP-1-derived macrophages upon 0 h and 48 h of MTB infection. (Data presented as mean ± SD; *n* = 4, at least three independent experiments with four replicates each; * *p* ≤ 0.05 was considered as statistically significant; ns, not significant).

We previously found that gliotoxin (**1**), 12, 13-dihydroxy-fumitremorgin C (**2**), and helvolic acid (**3**) exhibited direct anti-tuberculosis effects [5]. To further investigate how these compounds affect MTB infection in macrophages, we first detected the cytotoxicity of these compounds on THP-1-derived macrophages. A CCK8 kit was used to measure the cell proliferation upon treatments of different compounds at various concentrations. Isoniazid (INH) was used as a control. We found that both gliotoxin (0.5 μM and 1 μM) and 12,13-dihydroxy-fumitremorgin C (25 μM) inhibited cell proliferation, suggesting cytotoxicity. However, helvolic acid (25 μM) and isoniazid (10 μM) did not affect cell proliferation (Figure 1A–D). To exclude the possibility that anti-MTB effects of compounds were due to their cytotoxicity on THP-1 macrophages, we applied 0.25 μM gliotoxin, 10 μM 12, 13-dihydroxy-fumitremorgin C, 25 μM helvolic acid, and 10 μM isoniazid, respectively. Colony-forming units (CFU) assay showed that gliotoxin significantly decreased MTB infection by 83.63 ± 22.83% (mean ± SD, *n* = 4, *p* ≤ 0.05) at 48 h post infection, and the counts corresponded to MTB in infected THP-1 macrophages. However, 12, 13-dihydroxy-fumitremorgin C only decreased MTB infection by 29.14 ± 4.76% (mean ± SD, *n* = 4, *p* ≤ 0.05) at 48 h post infection. Helvolic acid displayed no apparent effect on MTB infection at 48 h. As a positive control, isoniazid remarkably inhibited MTB infection in macrophages at 48 h post infection. All these compounds did not affect MTB infection at 0 h of infection in THP-1 macrophages (Figure 1E,F). These results indicate that gliotoxin significantly suppressed MTB infection in the absence of cytotoxicity in THP-1-derived macrophages. 

### 2.2. Gliotoxin Suppresses MTB Infection in RAW 264.7 

**Figure 2:**
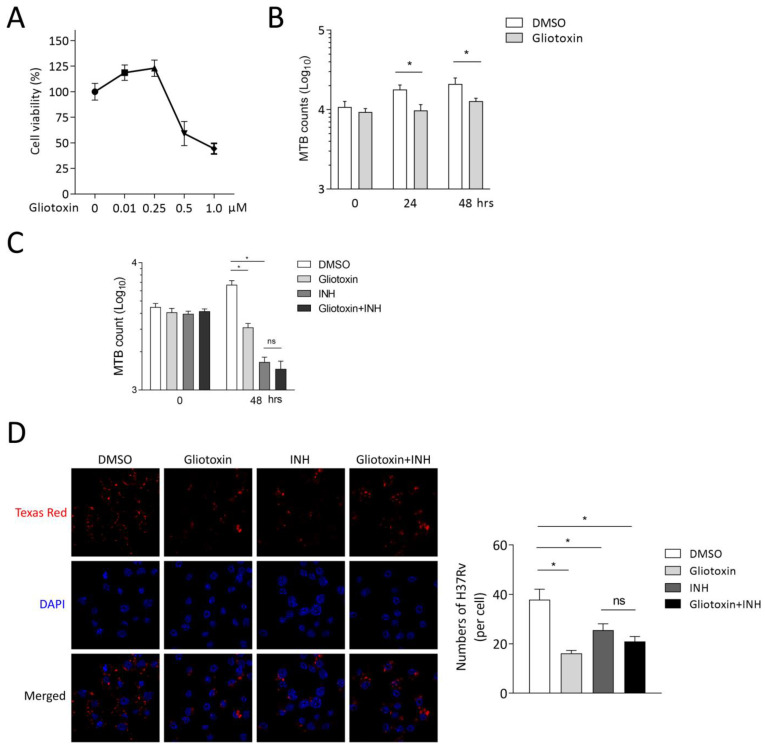
Gliotoxin suppressed MTB infection in RAW 264.7. (**A**) CCK8 assay showed the cytotoxicity of different concentrations of gliotoxin to RAW264.7 cells for 48 h. (**B**) Treating RAW264.7 cells with 0.25 μM gliotoxin, the intracellular mycobacterial viability was determined using CFU assay at 24 h and 48 h post infection. (**C**,**D**) RAW264.7 cells were treated with 0.25 μM gliotoxin, 1 μM isoniazid or the combination, CFU assay, and bacteria were stained with Texas red inside macrophages, with laser confocal microscopy assay applied. (Data presented as mean ± SD; *n* = 4, at least three independent experiments with four replicates each; * *p* ≤ 0.05 was considered as statistically significant; ns, not significant).

Mouse-macrophage-leukemia cell line RAW 264.7 was applied to further identify the effect of gliotoxin on MTB infection. RAW 264.7 was treated with different concentrations of gliotoxin for 48 h, and CCK8 assay showed that 0.25 μM gliotoxin exerted no cell cytotoxicity (Figure 2A). Then, CFU assay showed that 0.25 μM gliotoxin significantly reduced intracellular MTB loads by 45.19 ± 17.83% (mean ± SD, *n* = 4, *p* ≤ 0.05) at 24 h of infection and by 39.58 ± 11.87% (mean ± SD, *n* = 4, *p* ≤ 0.05) at 48 h of infection. Gliotoxin did not affect MTB infection at 0 h of infection in RAW 264.7 (Figure 2B). Isoniazid is the first-line antibiotic against MTB infection in clinic, and we found that 1 μM isoniazid significantly reduced MTB infection using CFU assay. However, Gliotoxin did not promote Mtb reduction with isoniazid treatment (Figure 2C). Then, we counted stained bacteria inside macrophages with laser confocal microscopy assay. The results showed that gliotoxin and isoniazid could significantly inhibit Mtb infection. Consistently, gliotoxin did not further reduce MTB infection under the treatment of isoniazid (Figure 2D). These results indicate that gliotoxin exerts significant anti-TB effect in mice macrophages. 

### 2.3. Gliotoxin Promotes Autophagy upon MTB Infection 

**Figure 3:**
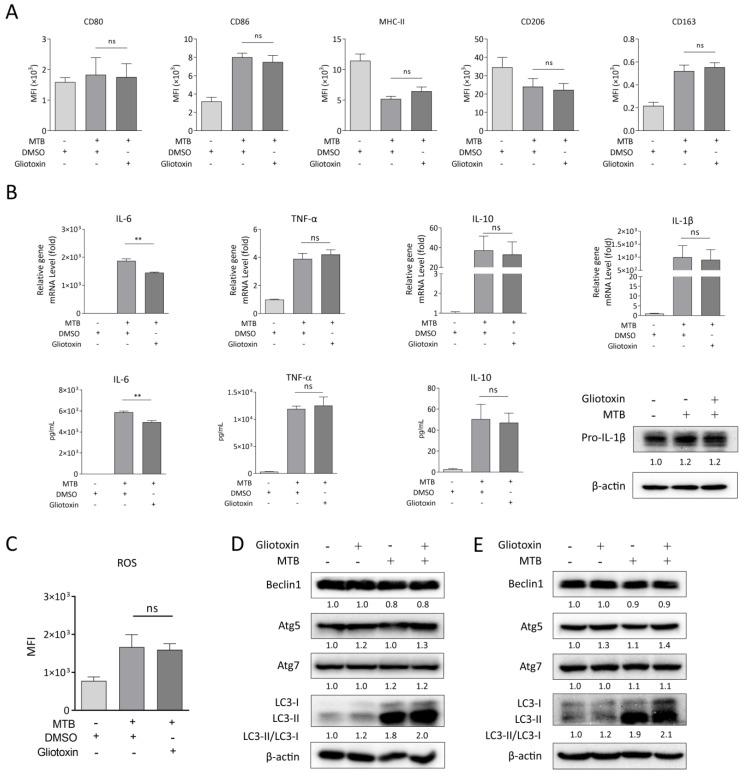
Gliotoxin promoted autophagy upon MTB infection. (**A**) M1 macrophage surface markers CD80, CD86, MHC-II, and M2 macrophage surface markers CD163, CD206 expression using flow cytometry analysis and (**B**) cytokines of IL-6, IL-1β, TNF-α, and IL-10 mRNA and protein expression were detected using qRT-PCR, Western blot, or ELISA analysis with gliotoxin treatment for 24 h in MTB-infected RAW264.7 cells. β-actin served as an internal reference. (**C**) ROS production was determined using an ROS Activity Assay Kit, and (**D**,**E**) ratio of LC3-II/LC3-I and Beclin1, Atg5, Atg7 expression were detected using Western blot assay with gliotoxin treatment for 24 h in MTB-infected RAW264.7 cells and THP-1 macrophages. β-actin served as an internal reference. (Data presented as mean ± SD; *n* = 3–4; ** *p* ≤ 0.01 was considered as statistically significant; ns, not significant.).

As we know that macrophage polarization, cytokines production, ROS generation, and autophagy machinery play important roles in regulating host immune response against MTB infection [15], we first detected the expression of M1-like macrophage surface markers (CD80, CD86, and MHC-II) and M2-like macrophage surface markers (CD163 and CD206) in MTB-infected RAW 264.7 with gliotoxin treatment using a flow cytometry analysis. We found that gliotoxin treatment did not affect surface-marker expression of M1-like or M2-like macrophages with MTB infection (Figure 3A). Next, we detected the expression of cytokines of IL-6, IL-1β, TNF-α, and IL-10 in MTB-infected RAW 264.7. We observed that mRNA expression of IL-1β, TNF-α, and IL-10 was not influenced by gliotoxin treatment in MTB-infected RAW 264.7. Consistently, TNF-α and IL-10 production and intracellular IL-1β expression were not affected by gliotoxin treatment (Figure 3B). However, gliotoxin significantly decreased IL-6 mRNA expression and production in MTB-infected RAW 264.7 (Figure 3B). ROS production was also not influenced by gliotoxin treatment during infection (Figure 3C). To investigate whether gliotoxin treatment regulates MTB infection through autophagy, we detected autophagosomal marker LC3 expression and autophagy-related protein expression including Beclin1, Atg5, and Atg7 in RAW 264.7 and THP-1 macrophages using Western blot analysis. The results showed that gliotoxin treatment promoted the expression of LC3 and Atg5, but not Beclin1 or Atg7 in MTB-infected RAW 264.7 and THP-1 macrophages (Figure 3D,E). These results indicated that the gliotoxin might inhibit MTB infection through autophagy, but not macrophage polarization, cytokines expression and production, or ROS generation in MTB-infected macrophages.

### 2.4. Gliotoxin Inhibits MTB Infection by Promoting Autophagy Machinery

**Figure 4:**
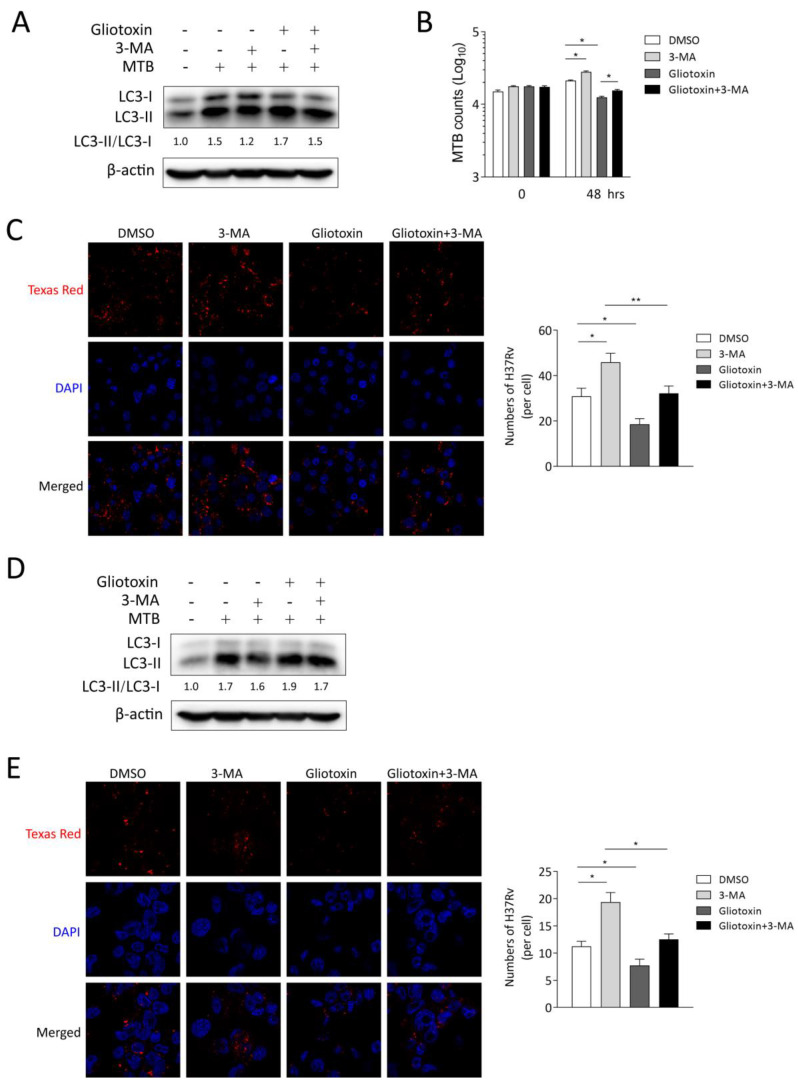
Gliotoxin inhibited MTB infection by promoting autophagy machinery. (**A**) Ratio of LC3-II/LC3-I was detected using Western blot assay with gliotoxin treatment or 3-MA treatment upon 24 h of MTB infection in RAW264.7 cells. β-actin served as an internal reference. (**B**) The intracellular bacterial load was determined using CFU assay and (**C**) laser confocal microscopy assay with gliotoxin, 3-MA or their combination at 48 h post infection. (**D**) Ratio of LC3-II/LC3-I was detected using Western blot assay with gliotoxin treatment or 3-MA treatment upon 24 h of MTB infection in THP-1 macrophages. β-actin served as an internal reference. (**E**) Texas red-stained bacteria in THP-1 macrophages with gliotoxin, 3-MA or their combination at 24 h post infection were detected using laser confocal microscopy assay. (Data presented as mean ± SD; *n* = 4; at least three independent experiments each with four replicates; * *p* ≤ 0.05; ** *p* ≤ 0.01 were considered as statistically significant).

To further investigate whether gliotoxin-promoted autophagy is the main cause of suppressing MTB infection in macrophages, we applied autophagy-inhibitor 3-methyladenine (3-MA) to treat RAW 264.7 and THP-1 macrophages. The results showed that 3-MA treatment inhibited autophagosomal marker LC3 expression and blocked gliotoxin-induced LC3 expression in RAW 264.7 and THP-1 macrophages (Figure 4A,D). 

CFU assay showed that 3-MA could significantly increase MTB infection, and partially restore bacterial loads inhibited by gliotoxin in RAW 264.7 (Figure 4B). Consistently, 3-MA treatment significantly increased intracellular bacteria and restored bacterial loads inhibited by gliotoxin using laser confocal microscopy assay in RAW 264.7 and THP-1 macrophages (Figure 4C,E). These results indicated that gliotoxin suppresses MTB infection through activating autophagy machinery in macrophages.

## 3. Discussion

MTB infection caused TB to be declared a global health emergency. It remains one of the world’s deadliest infectious diseases. Until now, isoniazid, rifampicin, ethambutol, and pyrazinamide have been recognized as first-line drugs to treat TB in clinics [16]. Development of MDR-TB, XDR-TB, and the emerging adverse effects associated with the first-line or second-line antitubercular drugs demand for urgent discovery of novel compounds against MTB infection [17]. Natural products and their derivatives have historically been recognized as sources of therapeutic agents, due to their characteristics of high chemical diversity and biochemical specificity [18]. In recent decades, accumulated studies have revealed that natural products play vital roles in curing different diseases including neurological disorders [19], diabetes [20], tumors [21], and infectious diseases, especially TB [17,22]. For example, a number of hydroethanolic total extracts from medicinal plants exerted comprehensive in vitro antimycobacterial activity in a human-fetal lung-fibroblast cell line of MRC-5 [23]. Recently, a growing number of anti-TB agents from marine sources such as fungi and marine organisms have received much attention, as the marine environment represents unique resources enclosing a massive biological diversity [24]. Five compounds extracted from the marine sponge *Haliclona* sp. were found with properties against latent MTB isolates [25]. In our previous study, we obtained 12 compounds from the cultures of the deep-sea-derived fungus *Aspergillus* sp. SCSIO Ind09F01. Among them, gliotoxin (**1**), 12, 13-dihydroxyfumitremorgin C (**2**), and helvolic acid (**3**) exerted anti-tuberculosis activities with the prominent MIC_50_ values of <0.03, 2.41, and 0.894 μM, respectively. Interestingly, gliotoxin showed great efficacy against MTB infection at a very low dose and its effect was even stronger than that of isoniazid [5]. However, the mechanism of action of all mentioned compounds in MTB-infected host cells is unknown. Therefore, it is vital to investigate how these drugs interact with host immune systems and respond to MTB infection [26]. Here, we have demonstrated that gliotoxin (**1**), 12, 13-dihydroxyfumitremorgin C (**2**), and helvolic acid (**3**) treatments displayed different effects on MTB infection without cytotoxicity. Helvolic acid (25 μM) showed no anti-MTB effect and 12, 13-dihydroxyfumitremorgin C (10 μM) possessed a weak anti-MTB effect. However, a low concentration of gliotoxin (0.25 μM) showed a potent effect on MTB infection in THP-1-derived macrophages. These discrepancies of their anti-TB effects between in vitro research and direct anti-TB studies demonstrate that the mechanism of how drug treatment regulates host response to MTB infection is essential to the development of novel drugs against TB. Although gliotoxin is a non-ribosomal peptide discovered to be mainly produced by *Aspergillus fumigatus* [27], we explored whether it could be a secondary metabolite from the deep-sea fungus *Aspergillus* sp. SCSIO Ind09F01. In the whole-cell screening of a diverse collection of small molecules as a method for generating anti-TB drugs, gliotoxin was found as a critical inhibitor, but they failed to generate mutants of MTB that were resistant to gliotoxin, indicating that gliotoxin may have a non-specific mechanism of action [28]. This encourages us to further investigate how gliotoxin interacts with host cells against MTB infection.

As previously mentioned, the traditional first line of anti-TB treatment regimens are extremely long and have side effects; it is necessary to investigate host-directed therapy to overcome the limitations of current anti-TB therapy, including prolonged treatment duration and drug toxicity [29]. Host-directed therapy has been well applied to treat TB that can target numerous host biological pathways [30]. These pathways include components/pathways of the immune system such as the phenotypic diversity of macrophages, cytokine production, ROS generation and autophagy, and could be promising targets for developing new anti-TB drugs [31,32]. However, in our study, we found that gliotoxin treatment only promoted autophagy machinery, but not phenotypic diversity of macrophages, cytokine production, or ROS production in MTB-infected macrophages. Therefore, gliotoxin suppressed MTB infection through autophagy in macrophages.

During MTB infection, autophagy is essential for mounting an effective host immune effector. In response to invading mycobacteria, the host innate immune response including cytokine and ROS production could activate autophagy [33]. Interferon (IFN)-inducible effector proteins, including immunity-related GTPase family M (IRGM) proteins, participate in host immune response to mycobacterial infection through IFN-γ-induced or conventionally induced (by rapamycin or starvation) autophagy in human macrophages [34]. We previously identified that IL-36γ activated Wnt5A and COX-2 to regulate autophagy and eliminate intracellular bacteria in macrophages [35,36]. NOX2-generated ROS plays an important role in LC3 recruitment to phagosomes and the subsequent clearance of the mycobacterial infection [37]. In addition, potential anti-TB reagents that targeted several host intracellular signaling pathways are involved in the activation of autophagy during MTB infection. For example, vitamin D3 and activation of vitamin D-receptor signaling led to the induction of cathelicidin through antibacterial autophagy to defend against MTB in human monocytes/macrophages [38]. Moreover, the roles of nuclear receptors agonists in antibacterial autophagy have been emphasized. Nuclear receptor subfamily 1, group D, member 1 (NRD1) activated autophagy and lysosomal biogenesis through increase of MAP1LC3-II and LAMP1 [39]. Peroxisome proliferator-activated receptor α (PPARα) and its agonists can upregulate colocalization of MTB phagosomes and autophagosomes, as well as lysosomal biogenesis to combat MTB infection in macrophages [40]. Estrogen-related receptor α (ERRα) is required for the transcriptional activation of autophagy-related genes in macrophages in response to various autophagy inducers [41]. Additionally, calcium-mobilizing agents, AICAR as AMPK activator and resveratrol (RSV) as SIRT1 activator could activate autophagy to promote the antimicrobial effects against MTB infection [14]. These findings emphasize that host autophagy plays a critical role in restricting mycobacterial pathogens. Based on this, several studies with high-throughput approaches have identified that numerous synthetic compounds could target autophagy to enhance antimicrobial defense against MTB infection [14]. Although we have identified that gliotoxin exerted a significant anti-TB effect in macrophages via autophagy machinery, the molecular mechanisms and regulatory factors of gliotoxin in modulating antibacterial autophagy need further investigation.

## 4. Materials and Methods

### 4.1. Cell Culture

THP-1 cells (human acute monocytic leukemia cells) and RAW264.7 cells were purchased from CELLCOOK (CC1904, Guangzhou, China). They were cultured with RPMI 1640 (Corning, NY, USA) or DMEM (Corning, NY, USA) in the presence of 10% FBS; the cells were maintained at an appropriate cell concentration of 3 × 10^5^ cells/mL. THP-1 cells were induced into a macrophage-like phenotype through treatment with 100 ng/mL phorbol myristate acetate (PMA, Pepro Tech, Cranbury, NJ, USA) for 48 h for further experimentation. At the same time, RAW264.7 cells are counted after resuspension for the next experiment.

### 4.2. Reagents

Gliotoxin (**1**), 12, 13-dihydroxyfumitremorgin C (**2**), and helvolic acid (**3**) were obtained as previously described [5]. They were dissolved in DMSO (Sigma-Aldrich, Saint Louis, MO, USA) and stocked at –80 °C. Isoniazid (Taiji Southwest Pharmaceutical Co. Ltd, Guangzhou, China) was dissolved in a culture medium at a concentration of 100 mM and stocked at –80 °C. 3-MA was dissolved in DMSO and stocked at –20 °C with a concentration of 1 M and a 2 h pretreatment of 5 mM. 3-MA was used to treat macrophages and detect for CFU assay and Western blot analysis.

### 4.3. CCK-8 Assay

We used a TransDetect^®^ Cell Counting Kit (FC101-03) (Transgen, Beijing, China) to measure proliferation of THP-1-derived macrophages and RAW264.7 cells. Cells were seeded with 3 replicate wells in 24-well culture plates (CS016-0128, ExCell Bio, Shanghai, China) in a volume of 500 μL per well at 5 × 10^4^ per well. Cell culture medium containing different concentrations of compound was added to each well for 48 h of incubation. Then, the medium was changed with a fresh medium including 10% CCK8 reagent and incubated for 2 h. A microplate spectrophotometer was used to detect the absorbance of the solution of each well at 450 nm. We calculated the cell survival rate according to the following formula: cell survival rate (%) = [A450 (sample value)/A450 (control mean value)] × 100.

### 4.4. Colony-Forming Units (CFU) Assay

*Mycobacterium tuberculosis* H37Rv (American Type Culture Collection) were collected through low-speed centrifugation (3000 rpm.) from 7H9 (broth, Becton Dickinson, Franklin, NJ, USA) liquid nutrient medium. The mycobacteria were suspended with DMEM or RPMI (10% FBS) and transferred into a grinder to generate single-bacterial suspension. The density of bacteria was measured with a spectrophotometer in OD_600_ (Tecan spark, Grödig, Austria). Bacteria infected macrophages at MOI = 5 for 1 h, and the cells were seeded in 12-well culture plate (THP-1 derived macrophages: 5 × 10^5^/well; RAW264.7 cells: 2 × 10^5^/well). Surplus bacteria were removed and washed with PBS three times. Cells continued to be cultured with a fresh medium or a medium containing a different compound. Then, they were lysed at the indicated time points with 0.01% Triton X-200 (Solarbio, Beijing, China). Lysates were transfer into 7H10 agar plates containing 10% OADC (Becton Dickinson, Franklin, NJ, USA) in serial dilution. Bacteria colonies’ statistics were obtained after the plates were incubated within an incubator for 3–4 weeks. All infections were performed in triplicate. 

### 4.5. RNA Extraction and Real-Time Quantitative Reverse-Transcription Polymerase Chain Reaction (qRT-PCR)

Total RNA of macrophages was extracted using a total RNA Kit I (200) (OMEGA, Norcross, GA, USA) according to the manufacturer, and 1000 ng of mRNA were reverse-transcribed into cDNA using reverse transcription kit Honor™II 1st Strand cDNA Synthesis SuperMix for qPCR (gDNA digester plus) (Novogene, Beijing, China). The expression of the cRNA level of the related gene was detected using a Unique AptamerTM qPCR SYBR^®^ Green Master Mix (No Rox) (Novogene, Beijing, China) on a LightCycler 480 thermocycler (Roche, Basel, Switzerland). The procedure of qRT-PCR includes an initial step at 95 °C for 120 s, followed by 35 cycles of amplification and quantification (95 °C for 15 s, 65 °C for 15 s, 68 °C for 20 s). The primer sequences used in this study are shown in Supplementary Table S1.

### 4.6. Western Blot Analysis

After washed with PBS, cells were treated with protein lysis buffer [41.6 mM SDS (Zhuosheng Biotech, Shanghai, China), 455 mM Tris HCl (pH 6.8) (Sangon Biotech, Shanghai, China), 26.9 μM, 30% (*v*/*v*) glycerol (SIGMA, St. Louis, MO, USA), and 10 μM DLDithiothreitol (DTT) (SIGMA, St. Louis, MO, USA)]. Protein samples were heated at 100 °C for 10 min and samples were subjected to sodium dodecylsulfate-polyacrilamide gel electrophoresis (SDS-PAGE) and transferred to polyvinylidene difluoride (PVDF) membranes (Merck KGaA, Darmstadt, Germany). The membranes were blocked with PBS-Tween 20 (0.1%) (GHTECH, Guangzhou, China) that contain 5% (*w*/*v*) BSA (Sigma, St. Louis, MO, USA), incubated with primary antibodies of LC3-I/II, pro-IL-1β, Beclin1, Atg5, Atg7 (Cell Signaling Technology, Danvers, MA, USA) (1:1000) with 5% (*w*/*v*) BSA at 4 °C overnight, with light shaking, incubated with HRP-conjugated secondary antibodies (Cell Signaling Technology, Danvers, MA, USA) (1:2000) for 1 h at room temperature after washing with PBS-Tween 20 (0.1%) three times × 10 min. Immunoreative bands were visualized in the FluorChem Systems (ProteinSimple, SiliconValley, CA, USA) after the addition of luminol-based chemiluminescent substrate (ECL, Thermo Fisher Scientific, Waltham, MA, USA). The integrated density of blotting bands was quantitatively analyzed using Image J FIJI software (National Institutes of Health) and normalized to 1.0 with β-actin as a control.

### 4.7. Reactive-Oxygen-Species (ROS) Detection

The cells were replaced with fresh culture medium in 6 wells with a plate density of 5 × 10^5^/well. MTB infected cells of MOI = 10 for 6 h. ROS Activity Assay Kit (Beyotime, Shanghai, China) was used to measure ROS. Cells were incubated with ROS probe DCFH-DA at concentrations of 10 µM for 30 min at 37 °C after cells were washed with DMEM three times. Then, we suspended the cells into a tube with PBS (1% FBS), and analyzed the fluorescence using BD LSRII Fortessa X-20 (BD Biosciences, Franklin, NJ, USA). Data analysis was performed using FlowJo v10.0 software (Tree Star, Ashland, OR, USA).

### 4.8. Confocal Laser Scanning Microscopy Analysis

Cells were suspended and counted from a Petri dish medium (10% FBS). Bacteria constantly infect cells (MOI = 3) in the density of 2 × 10^5^ for 24 h and were stained with Texas Red (Thermo Fisher Scientific, Waltham, MA, USA). Cells were fixed and permeated with 4% Paraformaldehyde and 0.2% Triton-X PBS. The nucleus was stained with 4, 6-diamidino-2-phenylindole (DAPI) (Sangon Biotech, Shanghai, China). Images were obtained with a Zeiss Axiovert LSM 880 instrument (Zeiss, Gottingen, Germany) Confocal laser scanning microscope. The number of bacteria were analyzed and counted from a Binary Image that was transformed from a Gray Scale Image of bacteria in Image J FIJI software. We counted at least 200 cells for each experiment.

### 4.9. Flow Cytometry (FCM) Analysis

For surface staining, the RAW264.7 with MTB infection for 24 h were harvested, washed, and stained for 30 min on ice with mixtures of fluorescently conjugated mAbs or isotype-matched controls. Cells were collected at a density of 5 × 10^5^, and suspended in staining buffer (1% FBS) with APC-anti-CD86, V450-anti-CD80, PE-anti-MHC-II, Percp eFluor 710-anti-CD163, PE-Cy7-anti-CD206 (eBioscience, Carlsbad, CA, USA) at 4 °C for 30 min. Then we washed the cells with buffer (1% FBS) three times after being fixed in 1% paraformaldehyde for 15 min. M1 and M2 macrophage markers were analyzed using flow cytometry on a flow cytometer (BD LSRII Fortessa X-20). Data were acquired as the fraction of labeled cells within a live-cell gate and analyzed using FlowJo v10.0 software. All gates were set on the basis of isotype-matched control antibodies.

### 4.10. Enzyme-Linked Immunosorbent Assay (ELISA) Analysis

The cell culture supernatant was harvested and centrifuged at 2000 rpm for 5 min. Cytokines (IL-6, TNF-α, and IL-10) were detected using an ELISA kit (ExCell Bio, Shanghai, China) according to the manufacturer’s protocol. The concentration of cytokines was measured using a Spectrophotometer (Tecan spark, Grödig, Austria).

### 4.11. Statistical Analysis

All the results were shown as mean values ± SD of at least triplicate experiments. Statistical analyses were performed using Mann–Whitney U test (GraphPad version 7). * *p* ≤ 0.05 and ** *p* ≤ 0.01 were considered as statistically significant.

## 5. Conclusions

In summary, we discover gliotoxin, a natural product from the deep-sea-derived fungus *Aspergillus* sp. SCSIO Ind09F01, is an anti-TB compound in macrophages. Mechanistic study has demonstrated that gliotoxin leads to the enhancement of autophagy to combat MTB infection. The well-established autophagy-inhibitor 3-MA could restore the decreased MTB infection with gliotoxin treatment. This research suggests gliotoxin is a new therapeutic product targeting autophagy during MTB infection in macrophages, and that gliotoxin could be a potential lead compound for the development of drugs against TB.

## Data Availability

The data presented in this study are available on request from the corresponding author.

## Supplementary Materials

The following supporting information can be downloaded at https://www.mdpi.com/article/10.3390/md21120616/s1, Table S1: List of mouse genes and corresponding primers for qRT-PCR assay.
