# Supplementary material for: Marine-Fungi-Derived Gliotoxin Promotes Autophagy to Suppress Mycobacteria tuberculosis Infection in Macrophage
Source: Mar Drugs. 2023 Nov 28;21(12):616. doi: 10.3390/md21120616 (PMC10745037; doi:10.3390/md21120616)
Supplement: Supplementary file 1 [file marinedrugs-21-00616-s001.zip › marinedrugs-2710697-supplementary.pdf]

Table S1. List of mouse genes and corresponding primers for qRT-PCR assay.

No.	Gene symbol	Full name	Primers (5' - 3')
1	Actin		F: CATTGCTGACAGGATGCAGAAGG
			R: TGCTGGAAGGTGGACAGTGAGG
2	TNF-α	Tumor necrosis factor	F: GGTGCCTATGTCTCAGCCTCTT
			R: GCCATAGAACTGATGAGAGGGAG
3	IL-1ß	Interleukin 1 beta	F: TGGACCTTCCAGGATGAGGACA
			R: GTTCATCTCGGAGCCTGTAGTG
4	IL-6	Interleukin 6	F: TACCACTTCACAAGTCGGAGGC
			R: CTGCAAGTGCATCATCGTTGTTC
5	IL-10	Interleukin 10	F: CGGGAAGACAATAACTGCACCC
			R: CGGTTAGCAGTATGTTGTCCAGC
6	CD80		F: CCTCAAGTTTCCATGTCCAAGGC
			R: GAGGAGAGTTGTAACGGCAAGG
7	CD86		F: ACGTATTGGAAGGAGATTACAGCT
			R: TCTGTCAGCGTTACTATCCCGC
8	CD163		F: GGCTAGACGAAGTCATCTGCAC
			R: CTTCGTTGGTCAGCCTCAGAGA
9	CD206		F: GTTCACCTGGAGTGATGGTTCTC
			R: AGGACATGCCAGGGTCACCTTT

F: forward primer; R: reverse primer.
